# Texture analysis of iodine maps and conventional images for k-nearest neighbor classification of benign and metastatic lung nodules

**DOI:** 10.1186/s40644-020-00374-3

**Published:** 2021-01-26

**Authors:** Simon Lennartz, Alina Mager, Nils Große Hokamp, Sebastian Schäfer, David Zopfs, David Maintz, Hans Christian Reinhardt, Roman K. Thomas, Liliana Caldeira, Thorsten Persigehl

**Affiliations:** 1 Department of Diagnostic and Interventional Radiology, University of Cologne, Faculty of Medicine and University Hospital Cologne, Kerpener Straße 62, 50937 Cologne, Germany; 2 Else Kröner Forschungskolleg Clonal Evolution in Cancer, University Hospital Cologne, Weyertal 115b, 50931 Cologne, Germany; 3 Department of Radiology, Massachusetts General Hospital, 55 Fruit St, White 270, Boston, MA 02114 USA; 4 Mint Medical GmbH, Burgstraße 61, 69121 Heidelberg, Germany; 5 Clinic I of Internal Medicine, University Hospital Cologne, 50931 Cologne, Germany; 6 Department of Hematology and Stem Cell Transplantation, University Hospital Essen, University Duisburg-Essen, German Cancer Consortium (DKTK partner site Essen), Essen, Germany; 7 Department of Translational Genomics, Center of Integrated Oncology Cologne-Bonn, Medical Faculty, University of Cologne, 50931 Cologne, Germany

**Keywords:** Lung nodules, Staging, Diagnosis, Differentiation, Dual-energy CT, Spectral detector CT, Texture analysis, Lung metastases, Oncologic imaging

## Abstract

**Background:**

The purpose of this study was to analyze if the use of texture analysis on spectral detector CT (SDCT)-derived iodine maps (IM) in addition to conventional images (CI) improves lung nodule differentiation, when being applied to a k-nearest neighbor (KNN) classifier.

**Methods:**

183 cancer patients who underwent contrast-enhanced, venous phase SDCT of the chest were included: 85 patients with 146 benign lung nodules (BLN) confirmed by either prior/follow-up CT or histopathology and 98 patients with 425 lung metastases (LM) verified by histopathology, ^18^F-FDG-PET-CT or unequivocal change during treatment. Semi-automatic 3D segmentation of BLN/LM was performed, and volumetric HU attenuation and iodine concentration were acquired. For conventional images and iodine maps, average, standard deviation, entropy, kurtosis, mean of the positive pixels (MPP), skewness, uniformity and uniformity of the positive pixels (UPP) within the volumes of interests were calculated. All acquired parameters were transferred to a KNN classifier.

**Results:**

Differentiation between BLN and LM was most accurate, when using all CI-derived features combined with the most significant IM-derived feature, entropy (Accuracy:0.87; F1/Dice:0.92). However, differentiation accuracy based on the 4 most powerful CI-derived features performed only slightly inferior (Accuracy:0.84; F1/Dice:0.89, *p*=0.125). Mono-parametric lung nodule differentiation based on either feature alone (i.e. attenuation or iodine concentration) was poor (AUC=0.65, 0.58, respectively).

**Conclusions:**

First-order texture feature analysis of contrast-enhanced staging SDCT scans of the chest yield accurate differentiation between benign and metastatic lung nodules. In our study cohort, the most powerful iodine map-derived feature slightly, yet insignificantly increased classification accuracy  compared to classification based on conventional image features only.

**Supplementary Information:**

The online version contains supplementary material available at 10.1186/s40644-020-00374-3.

## Background

Lung nodules are one of the most common incidental findings in chest computed tomography (CT) [1]. Different imaging features depicted in CT of the chest can be used to facilitate prediction of malignancy, especially large nodule size, part-solid appearance and/or spiculation [2–4]. While nodules in non- cancer patients are mostly benign, probability of malignancy is much higher when found in cancer patients at staging examinations [5]. However, for cancer patients, Fleischner criteria are not applicable [6]. Hence, cancer patients with ambiguous lung nodules often undergo either additional follow-up to detect size increase or biopsy of the referring lesions [7, 8]. The necessity for follow-up implies the risk of delayed diagnosis and additional radiation exposure, while biopsies may lead to periinterventional complications such as pneumothorax or pulmonary hemorrhage [9]. Furthermore, uncertainty regarding metastatic status may even alter therapy [10].

Several approaches have been suggested to investigate differentiation of lung nodules within one examination without further need for additional follow-up. Until now, the majority of referring studies were primarily focused on discrimination between benign lung nodules and primary lung cancer [11–15]. However, with regards to differentiation of benign lung nodules and lung metastases at contrast-enhanced staging CT, data is much sparser. As one of the approaches proposed to this regard, it has been revealed that first order texture features derived from contrast-enhanced chest CT scans could be a feasible method do distinguish between benign and metastatic lung nodules [16, 17]. Another technique that has been investigated in this setting was the application of dual-energy CT derived iodine maps [18]. These maps can be calculated based on the separate acquisition of Photoelectric and Compton-weighted datasets [19]. It has been shown that such maps may be advantageous for the purpose of classification of pulmonary nodules as they reflect lesion vascularity [20–22]. However, accuracies obtained with either of the two methods have not been sufficiently high to pave the way to clinical application.

Our hypothesis was that texture analysis and dual-energy CT-derived iodine maps may work synergistically to facilitate lung nodule differentiation; we focused on first order texture analysis which has been described to be more reproducible than higher-order features, which we assumed to be favorable when applying it to DECT data [23]. Consequently, the study purpose was to examine the differentiation between benign lung nodules and metastases based on first-order texture features obtained from spectral-detector-CT derived iodine maps and conventional CT images.

## Methods

### Patients

**Fig. 1:**
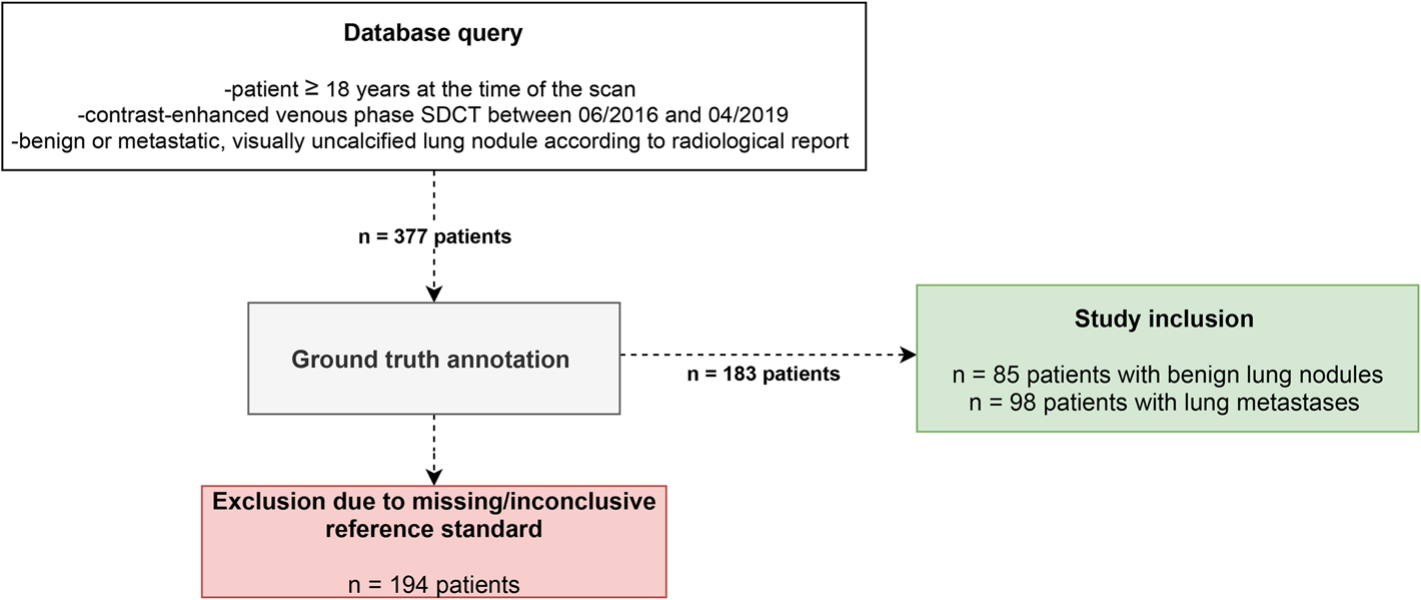
Workflow for inclusion and exclusion of patients

A retrospective database query was executed to identify oncologic patients (≥ 18 years) who underwent clinically indicated, contrast-enhanced, venous phase SDCT of the chest and who were diagnosed with visually uncalcified, metastatic or benign lung nodules according to the radiological report. Subsequently, lung nodules with a diameter equal or greater than 5 mm were selected by an experienced radiologist and all nodules were correlated with ground truth as indicated below; if ground truth was absent, patients were excluded. Figure 1 shows the workflow for inclusion and exclusion of study subjects.

### Ground truth correlation and lesion annotation

Before study inclusion, each lesion was correlated with a reference standard. For the subgroup of lung metastases, this was based either on

1. Histopathologic confirmation of metastatic disease **and/or**
2. Unequivocal radionuclide uptake in referring FDG-PET/CT examinations **and/or**
3. Unequivocal increase in size over the course of multiple follow-up examinations or change in size during anticancer treatment.

Eligibility criteria for benign lung nodules were:

1. Histopathologic confirmation of inflammation/post-inflammatory changes without malignancy **and/or**
2. Constant size without treatment compared to prior or follow-up CT for a period of at least 6 months, no history of lung metastases

### Image acquisition and reconstruction

All scans were performed on a clinical dual-energy CT scanner (IQon; Philips Healthcare, Best, the Netherlands) with the following scan parameters: supine patient position, inspirational breath hold, pitch=0.67; rotation time=0.33 s, collimation=64 × 0.625 mm; matrix=512 × 512; tube voltage=120 kVp; tube current modulation enabled (DoseRight 3D-DOM; Philips Healthcare). For acquisition of contrast-enhanced scans (mainly combined chest/abdomen examinations), all patients received a body weight–adapted bolus of iodinated contrast media (< 55 kg: 1 ml/kg; 55–120 kg: 100 ml; > 120 kg: 120 ml; Accupaque, 350 mg/mL; GE Healthcare, Chicago, IL) which was administered via a peripheral vein with a flow rate of 3.5 mL/sec and followed by a saline flush of 30 mL. Bolus-tracking technique with a delay of 50 s was enabled to receive venous phase scans of the chest (and abdomen, if clinically indicated). For reconstruction of conventional images (CI), a hybrid-iterative reconstruction algorithm was used (iDose 3, filter YA, Philips Healthcare) and standard lung window was chosen. Iodine maps (IM) were reconstructed using a dedicated spectral reconstruction method (Spectral, filter B, level 3, Philips Healthcare); 2 mm slice thickness and 1 mm section increment were chosen throughout all datasets.

### Post-processing

CT datasets were transferred to a proprietary software for oncologic follow-up (mint lesion research, Mint Medical GmbH, Heidelberg, Germany). Lung nodules were semi-automatically contoured based on CI. CI and IM were co-registered, and segmentations were transferred from CI to IM. After that, segmentations were double-checked in order to warrant consistent volumes of interest between both reconstructions. Volumetric Hounsfield unit attenuation (HU), iodine concentration (IC [mg/ml]) as well as first order texture features (entropy, kurtosis, mean of the positive pixels (MPP), skewness, uniformity and uniformity of the positive pixels (UPP)) within the referring volumes of interests from both datasets were obtained (supplementary Table 1).

### Pre-processing and feature analysis

Values for HU and IC and referring first order texture features were exported. Mean HU and IC between the two groups were compared. 15 benign lesions with a HU higher than one standard deviation above the mean HU were excluded due to suspected calcification.

Features were tested individually (scikit-learn 0.21.3) by means of area under the receiver operator characteristic curve (AUC), F-statistics and Mutual Information (MI).

### Multiparametric classification

Features were normalized (zero mean and unit variance) and data was transferred to a k-nearest neighbor (KNN) classifier with 10 neighbors using Euclidean distance metric. The classifier was then evaluated with 5-fold and Leave One-Out Cross Validation.

### Statistical analysis

Quantitative attenuation and iodine values were compared using Wilcoxon test. Statistical significance was determined as *p* ≤0.05. For feature testing, MI was calculated. For classification evaluation, F1 score, accuracy, specificity and sensitivity were computed (supplementary Table 2).

## Results

### Study cohort

**Fig. 2:**
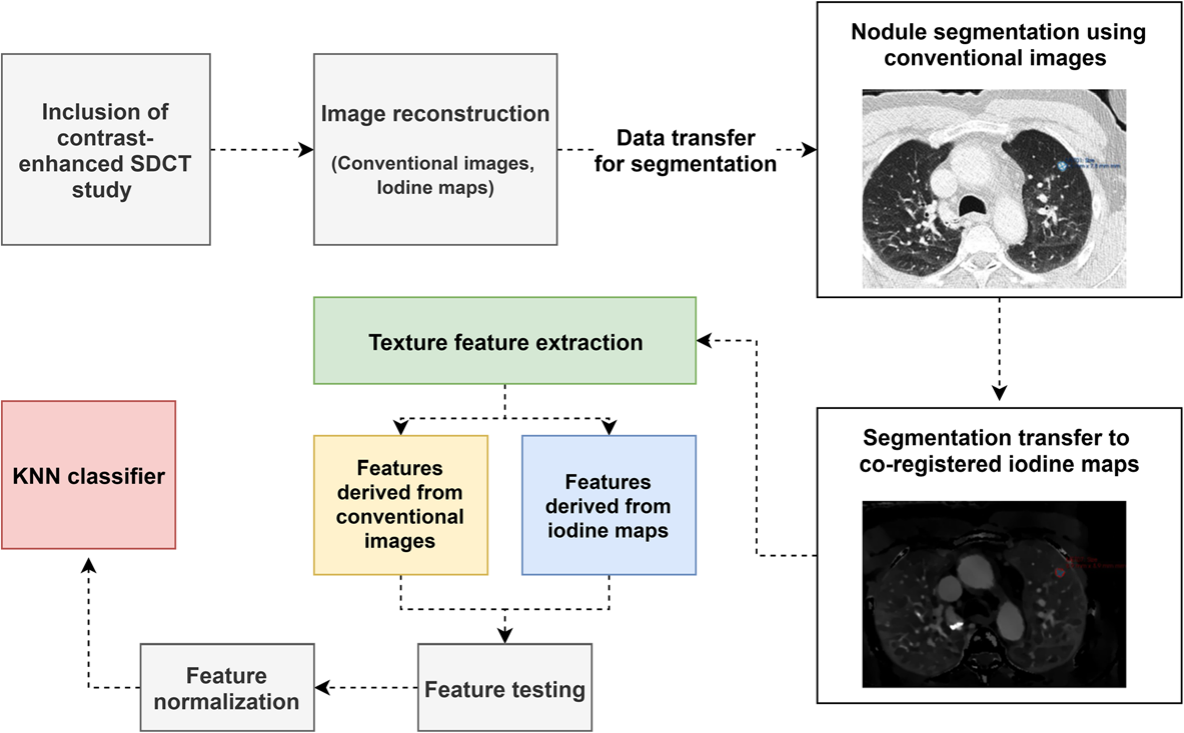
Methodological workflow from study inclusion and image reconstruction to image segmentation, feature extraction, testing and KNN-based classification

183 cancer patients (96 men and 87 women, mean age 63.2 ± 13.0) who underwent SDCT of the chest were included: 85 patients with 161 benign lung nodules and 98 patients with 425 lung metastases. Median time of available imaging follow-up for ground truth correlation was 22.5 months, ranging from 6 months to 79 months. Table 1 gives an overview on patient characteristics. 425 metastases and 146 benign lesions were used for training and testing comprising approximately 70 and 30% of the data, respectively. Accordingly, the training group was composed of 105 benign lesions and 294 metastases, while the testing group comprised 41 benign lesions and 131 metastases. Figure 2 gives an overview on the methodological workflow of the study.

**Table 1:** Patient characteristics

	Patients with benign lung nodules	Patients with lung metastases
*Patients*	85	98
*Patient demographics*		
*Sex (men/women)*	47/38	49/49
*Mean age (years)*	62.7 ± 12.2	63.7± 13.8
*Mean Dose (CTDIvol)*	14.3 ± 6.7	14.6 ± 6.9
*Underlying diseases*		
*Melanoma*	18	32
*Esophageal cancer*	8	5
*Sarcoma*	7	6
*Breast cancer*	4	9
*Lymphoma*	11	1
*Colorectal cancer*	3	9
*Pancreatic cancer*	6	5
*Renal cell cancer*	1	8
*Urothelial carcinoma*	2	5
*Testicular cancer*	4	1
*Liver cancer*	1	3
*Ovarian cancer*	0	4
*Other oncologic diseases*	16	10
*No oncologic diseases*	4	0
*Ground truth*		
*Follow-up*	80	80
*Pathology report*	5	17
*PET/CT*	0	1

### Monoparametric analysis

**Fig. 3:**
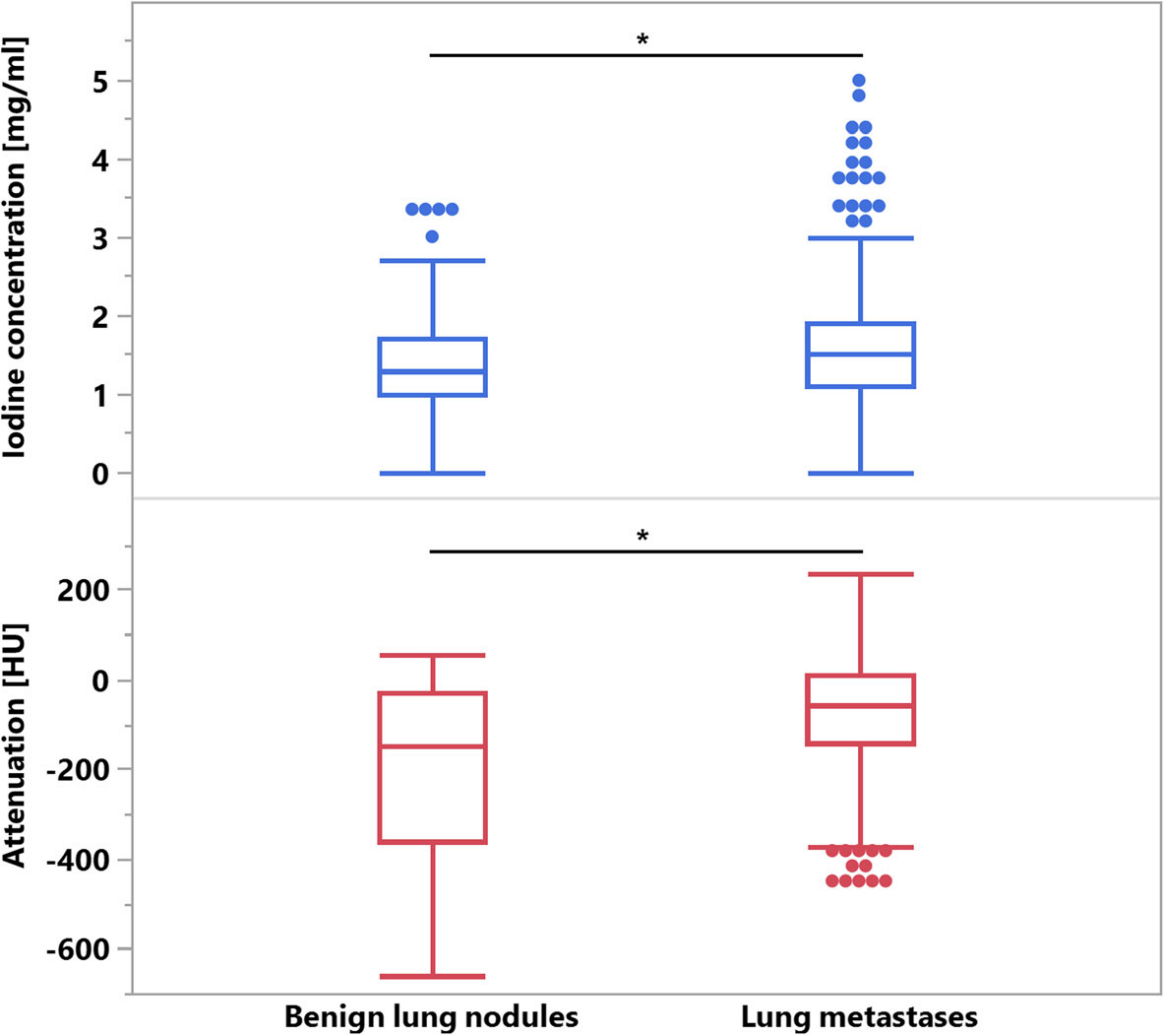
Attenuation and iodine concentration of benign lung nodules and lung metastases. Both were significantly higher in lung metastases yet overlap between the two lesion types was large

**Fig. 4:**
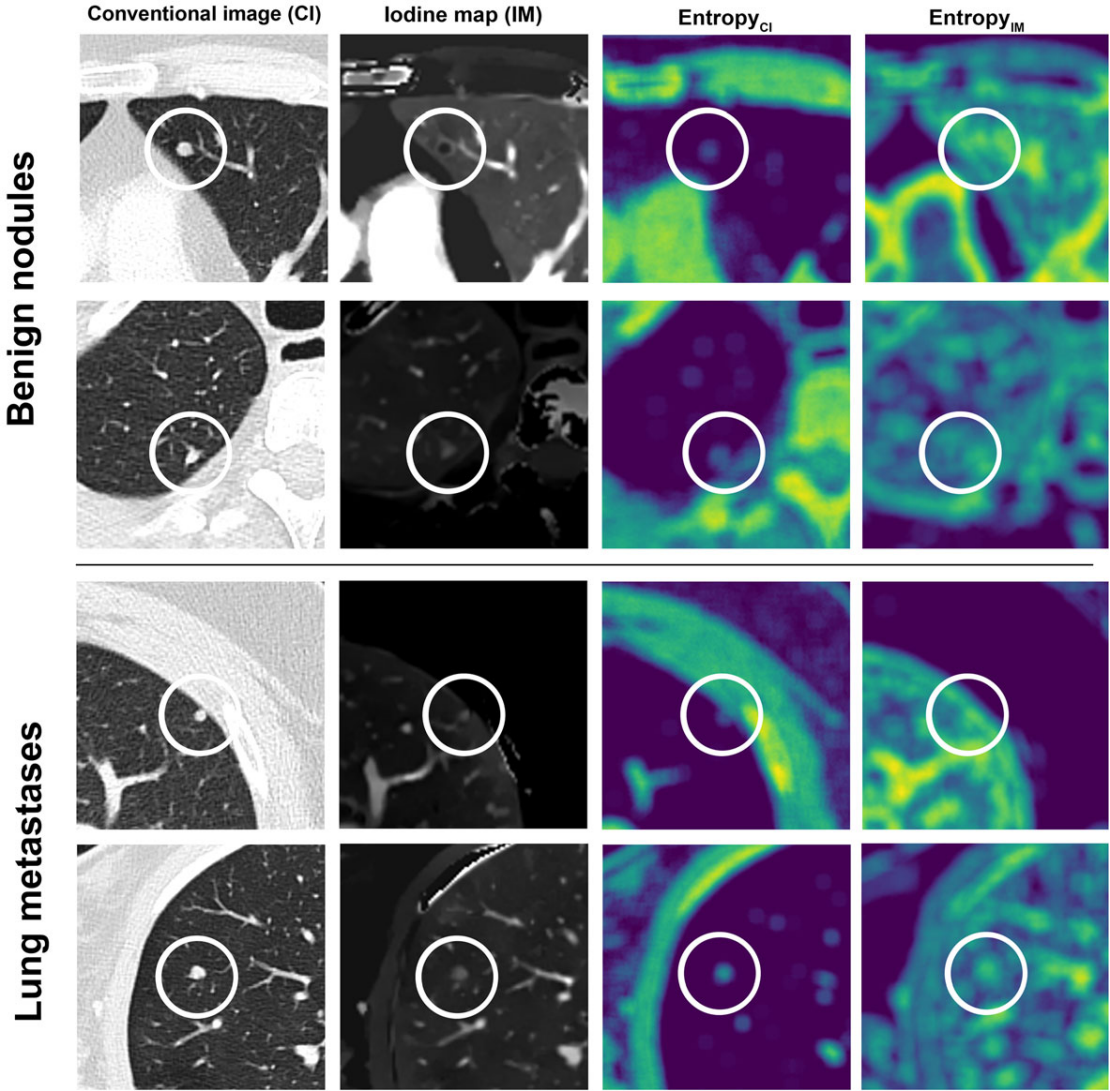
Examples of benign nodules as shown in conventional images (CI; left column), iodine maps (IM; second column from the left) and entropy texture maps derived from CI and IM (right columns). It is revealed that some benign and metastatic nodules may be clearly distinguished by means of their iodine uptake (top row vs bottom row) while some benign nodules show comparable iodine concentration values as metastases (second vs third row), hampering accurate differentiation

Hounsfield unit attenuation and iodine concentration were both significantly higher in metastatic (Attenuation: − 80.1 ± 192.0 HU; IC: 1.6 ± 0.5 mg/ml) than in benign lung nodules (Attenuation: − 170.5 ± 173.5 HU; IC: 1.4 ± 0.5 mg/ml; both *p*≤0.05). However, for both parameters, a significant data overlap was observed between the two lesion types (Fig. 3). Consequently, area under the ROC analysis revealed a low AUC of 0.67 and 0.58 for HU and IC, respectively, regarding benign and metastatic nodule differentiation. Pertaining to texture features, kurtosis, skewness and uniformity derived from CI showed significant differences between benign nodules and lung metastases, while for iodine map derived features, significant differences were found for entropy, kurtosis, uniformity and UPP; Table 2 shows the comparison of mean values of all tested features between benign and metastatic lung nodules. Figure 4 depicts exemplary cases of metastatic and benign lung nodules with entropy feature maps, which was the most powerful iodine map-derived feature.

**Table 2:** Mean values of all tested features for benign and metastatic lung nodules

	Benign lung nodules	Lung metastases	***p***-value
*Attenuation*_*CI*_	−170.49	−80.12	*p*≤0.05
*Attenuation SD*_*CI*_	173.54	191.96	*p*≤0.05
*Entropy*_*CI*_	6.70	8.01	*p*≤0.0001
*Kurtosis*_*CI*_	3.90	4.26	*p*≤0.0001
*MPP*_*CI*_	94.61	98.09	*p*=0.18
*Skewness*_*CI*_	−0.21	−0.80	*p*≤0.0001
*Uniformity*_*CI*_	0.01	0.01	*p*≤0.0001
*UPP*_*CI*_	0.00	0.00	*p*=0.63
*Iodine concentration*	1.44	1.58	*P*≤0.05
*Iodine concentration SD*	0.47	0.49	*P*=0.24
*Entropy*_*IM*_	4.90	5.21	*p*≤0.0001
*Kurtosis*_*IM*_	3.67	4.34	*p*≤0.0001
*MPP*_*IM*_	1.45	1.60	*p*≤0.05
*Skewness*_*IM*_	0.42	0.31	*p*=0.06
*Uniformity*_*IM*_	0.05	0.04	*p*≤0.0001
*UPP*_*IM*_	0.04	0.04	*p*≤0.0001

### Feature analysis

For feature analysis, only the training cohort was used. Individual feature testing identified entropy, uniformity and skewness as well as mean HU value derived from conventional images as the four most powerful features for lung nodule differentiation. The most powerful iodine map-derived feature was entropy. Table 3 includes AUC, F-statistics and MI values for each feature tested.

**Table 3:** Results from individual feature testing including area under the receiver operating characteristics curve (AUC), F1 score and mutual information (MI score)

	*AUC*	*F1 score*	*MI*
*Attenuation*	0.67	1.48 X 10^−12^	0.08
*Attenuation SD*	0.58	6.07 X 10^−03^	0.02
*Entropy*_*CI*_	0.83	7.45 X 10^−31^	0.17
*Kurtosis*_*CI*_	0.63	2.97 X 10^−01^	0.02
*MPP*_*CI*_	0.64	2.70 X 10^−7^	0.03
*Skewness*_*CI*_	0.74	3.05 X 10^−9^	0.07
*Uniformity*_*CI*_	0.82	7.75 X 10^−26^	0.15
*UPP*_*CI*_	0.56	1.41 X 10^−1^	0.11
*Iodine concentration*	0.58	1.06 X 10^−02^	0.00
*Iodine concentration SD*	0.52	4.71 X 10^−01^	0.00
*Entropy*_*IM*_	0.64	1.36 X 10^−02^	0.03
*Kurtosis*_*IM*_	0.62	1.68 X 10^−01^	0.00
*MPP*_*IM*_	0.58	9.74 X 10^−03^	0.00
*Skewness*_*IM*_	0.55	4.49 X 10^−01^	0.03
*Uniformity*_*IM*_	0.59	4.93 X 10^−01^	0.02
*UPP*_*IM*_	0.60	8.59 X 10^−01^	0.02

### Multiparametric classification

Applied to the training cohort, KNN using 5-Fold Cross Validation yielded optimal nodule differentiation when the best 4 CI-derived features were used (Accuracy:0.87; F1/Dice: 0.91). When combining the 4 best CI-derived features with the best IM-derived feature (Entropy_IM_), accuracy was on a comparable level (Accuracy:0.86; F1/Dice: 0.90).  

When using all iodine map-derived features without CI-derived features, classification accuracy was lower (Accuracy: 0.73; F1/Dice: 0.83). When applied to the testing cohort, KNN yielded the best nodule differentiation when using all CI-derived features and iodine-derived entropy (Accuracy:0.86; F1/Dice:0.91). Here, the 4 best CI-derived features yielded a lower accuracy, yet without statistical significance (Accuracy: 0.84, F1/Dice: 0.89). Tables 4 and 5 provide an overview on the results of KNN-based multiparametric classification in the training and testing cohort, respectively.

**Table 4:** Results of the training cohort. KNN-based lung nodule classification with 5-fold cross validation using different combinations of CI- and iodine-derived texture features

	Accuracy	F1 / Dice	Sensitivity	Specificity
4 best CI-derived features	0.87 ± 0.03	0.91 ± 0.02	0.94 ± 0.03	0.69 ± 0.06
4 best CI-derived features + Entropy_IM_	0.86 ± 0.04	0.90 ± 0.03	0.95 ± 0.04	0.65 ± 0.05
All CI-derived features	0.86 ± 0.02	0.91 ± 0.02	0.95 ± 0.02	0.64 ± 0.07
All iodine-derived features	0.73 ± 0.02	0.83 ± 0.02	0.92 ± 0.04	0.28 ± 0.08
All CI-derived features + Entropy_IM_	0.86 ± 0.02	0.90 ± 0.02	0.96 ± 0.01	0.61 ± 0.08
All features	0.83 ± 0.06	0.89 ± 0.04	0.95 ± 0.03	0.56 ± 0.12

**Table 5:** Results after application of the classifier to the test cohort showing lung nodule classification based on different combinations of CI- and iodine-derived texture features

	Accuracy	F1/Dice	Sensitivity	Specificity
*4 best CI-derived features*	0.84	0.89	0.89	0.69
*4 best CI-derived features + Entropy*_*IM*_	0.86	0.91	0.91	0.71
*All CI-derived features*	0.87	0.91	0.92	0.71
*All iodine-derived features*	0.74	0.84	0.90	0.24
*All CI-derived features + Entropy*_*IM*_	0.87	0.92	0.93	0.67
*All features*	0.84	0.90	0.93	0.52

## Discussion

This study evaluated differentiation between benign and metastatic lung nodules at staging spectral detector CT (SDCT) of the chest based on first-order texture features derived from quantitative iodine maps (IM) and conventional images (CI).

Distinguishing metastatic from non-metastatic lung nodules is a clinical scenario of high relevance. Our results suggest that the proposed method may help to improve M staging in the context of lung metastases which is important for determining prognostic outcome as well as therapeutic approaches of many oncologic diseases. Although lung metastases demonstrated a significantly higher attenuation and iodine concentration than benign nodules, the observed overlaps for both parameters between the two lesion types were high. As a result, nodule differentiation was poor when using either HU attenuation or iodine concentration exclusively. In contrast, first order texture analysis yielded highly accurate differentiation between benign and metastatic pulmonary nodules. Applied to the testing cohort, accuracy was highest for the combination of all CI-based texture features and the most powerful iodine-derived feature, entropy; yet without statistical significance compared to the differentiation based on CI-derived features only.

Increased iodine enhancement of lung nodules has been described as an indicator for both vascularity and malignancy [3, 24]. Hence, we expected that texture features derived from iodine maps would provide better insights to the vascular structure of lung nodules than conventional images, facilitating nodule differentiation; however, this hypothesis could not be confirmed by the results of our study. One possible reason may be that material separation between iodine and calcium as provided by dual-energy CT has been described to be limited in very small volumes [25]. Yet, despite this known limitation, we decided to include smaller nodules in our analysis as they are frequently encountered in staging CT of the chest and therefore play an important role in terms of clinical nodule differentiation [26]. To mitigate the effect of iodine/calcium-related misclassification, we only included patients having nodules without typical calcifications which can be considered a strong predictor for benign origin [27]. Further, during preprocessing, 15 additional nodules were excluded based on high HU values suggesting calcifications. However, smaller calcifications may still be present in many of the benign lesions included, contributing to the low specificity attained with iodine concentration and iodine map-derived texture parameters. Another explanation for the described results may be that iodine maps may not be a suitable data input for first order texture analysis as their generic appearance is blurrier compared to conventional images.

Our results must be put into context of the heterogeneity of our dataset. It comprised a wide range of underlying, heterogenic oncologic diseases. While this can be considered favorable in terms of generalizability, classification accuracy of metastases might vary depending on the underlying oncologic disease which is why validation of our proposed approach in larger datasets required. Moreover, while we refrained from including post-therapeutic metastatic residuals or metastases in the state of size decrease following therapy response, many of the included patients underwent chemotherapy. This might alter cellularity and vascularity of lung metastases, potentially changing feature characteristics; yet again, an ambiguous nodule in a patient with history of or currently under chemotherapy is a scenario that might also be encountered clinically which is why we think including such cases for model training improves its clinical applicability.

Although there are several studies that suggested an added value of iodine quantification for lung nodule characterization [18, 21, 28, 29], one recent study indicated that in conventional CT, contrast-enhancement did not improve texture analysis-based subclassification of lung adenocarcinoma [30] which supports the results we found. Regarding the use of texture analysis of conventional CT, our results are in line with previous studies that reported an accurate nodule differentiation: for differentiation of metastatic and benign lung nodules, Cho et al. reported an AUC of 0.86 [17]. Other studies particularly elucidated the potential of entropy and the absence of uniformity for differentiation between benign nodules and adenocarcinoma [15, 16] or prognostic evaluation of lung cancer [31]. In accordance with their results, entropy derived from conventional images was higher in pulmonary metastases in our study and it was the most powerful feature for nodule differentiation; also, it was the most powerful iodine-derived feature.

Our study is subject to several limitations that need to be addressed. First, we did not assess higher order texture features. As the combination of DECT-derived iodine maps and texture analysis has not been tested before for differentiation of benign and metastatic lung nodules, we wanted to focus on first order features as they were previously described as more robust compared to higher order features [23]; yet, subsequent studies with higher order features should be encouraged. Second, our study comprised a much larger number of lung metastases than benign nodules which introduces class imbalance. Third, histopathologic information was available only in a small proportion of nodules included which is owed to the limited number of patients that underwent biopsy especially in case of benign lung nodules. While the diagnosis of the metastatic nodules we included can be assumed to be relatively certain based on unequivocal changes in follow-up imaging and diagnosis of underlying diseases, the benign nodules often remained unspecified and will predominantly comprise granulomas, scar tissue from previous infections and hamartomas; despite long follow-up periods and refraining from including patients with known history of pulmonary metastases, a small number of non-vital metastatic residuals may have been present in this subgroup. However, this reflects the typical clinical scenario in oncological patients for which the non-invasive characterization is most important for individual optimized treatment planning. Last, most of the patients we included were diagnosed with oncologic diseases; this limits generalization to non-oncologic patients with low probability of lung nodule malignancy.

## Conclusions

To conclude, our study revealed that KNN classification based on first order texture features improves differentiation of benign and metastatic lung nodules in contrast-enhanced staging SDCT of the chest compared to mono-parametric differentiation. Texture features derived from iodine maps did not significantly improve differentiation compared to the same features obtained from conventional images. Further investigation of possible underlying technical limitations of SDCT as well as analysis of other material-specific maps for lung nodule differentiation should be pursued.

## Supplementary Information

**Additional file 1 Supplemental Table 1.** Calculation of first order texture features. X_*i*_ = signal intensity of a voxel; N = number of voxels in the region of interest (ROI). p_*j*_ = probability of intensity range j in the ROI; Ng = the number of discretized intensity values in the ROI. **Supplemental Table 2.** Calculation of mutual information (MI), F1-score, diagnostic accuracy, sensitivity and specificity.

## Data Availability

Please contact the corresponding author for data requests.

## Abbreviations

BLN: Benign lung nodule
CI: Conventional images
DECT: Dual energy CT
IM: Iodine maps
KNN: K-nearest neighbor classification
LM: Lung metastasis
SDCT: Spectral detector CT

## References

[1] McCarville MB, Lederman HM, Santana VM, Daw NC, Shochat SJ, Li C-S, Kaufman RA (2006). Distinguishing benign from malignant pulmonary nodules with helical chest CT in children with malignant solid tumors. Radiology.

[2] McWilliams A, Tammemagi MC, Mayo JR, Roberts H, Liu G, Soghrati K (2013). Probability of cancer in pulmonary nodules detected on first screening CT. N Engl J Med.

[3] Swensen SJ, Silverstein MD, Ilstrup DM, Schleck CD, Edell ES (1997). The probability of malignancy in solitary pulmonary nodules. Application to small radiologically indeterminate nodules. Arch Intern Med.

[4] Li F, Sone S, Abe H, MacMahon H, Doi K (2004). Malignant versus benign nodules at CT screening for lung cancer: comparison of thin-section CT findings. Radiology..

[5] Evangelista L, Panunzio A, Polverosi R, Pomerri F, Rubello D (2014). Indeterminate lung nodules in cancer patients: pretest probability of malignancy and the role of 18F-FDG PET/CT. AJR Am J Roentgenol.

[6] MacMahon H, Naidich DP, Goo JM, Lee KS, Leung ANC, Mayo JR (2017). Guidelines for Management of Incidental Pulmonary Nodules Detected on CT images: from the Fleischner society 2017. Radiology..

[7] Caparica R, Mak MP, Rocha CH, Velho PHI, Viana P, Moura MRL (2016). Pulmonary nodules in patients with nonpulmonary Cancer: not always metastases. J Glob Oncol.

[8] Larici AR, Farchione A, Franchi P, Ciliberto M, Cicchetti G, Calandriello L, et al. Lung nodules: Size still matters. Eur Respir Rev. 2017. 10.1183/16000617.0025-2017. 10.1183/16000617.0025-2017 PMC9488618 29263171

[9] Tai R, Dunne RM, Trotman-Dickenson B, Jacobson FL, Madan R, Kumamaru KK, Hunsaker AR (2016). Frequency and severity of pulmonary hemorrhage in patients undergoing percutaneous CT-guided transthoracic lung biopsy: single-institution experience of 1175 cases. Radiology..

[10] Rapicetta C, Lococo F, Davini F, Carleo F, Kauppi J, Di Stefano TS (2019). Is Adjuvant Chemotherapy Worthwhile After Radical Resection for Single Lung Metastasis From Colorectal Cancer? A Multicentric Analysis Evaluating the Risk of Recurrence. Front Oncol.

[11] Causey JL, Zhang J, Ma S, Jiang B, Qualls JA, Politte DG, et al. Highly accurate model for prediction of lung nodule malignancy with CT scans. Sci Rep. 8:1–12. 10.1038/s41598-018-27569-w. 10.1038/s41598-018-27569-w PMC6006355 29915334

[12] Dilger SKN, Uthoff J, Judisch A, Hammond E, Mott SL, Smith BJ (2015). Improved pulmonary nodule classification utilizing quantitative lung parenchyma features. J Med Imaging (Bellingham).

[13] Hawkins S, Wang H, Liu Y, Garcia A, Stringfield O, Krewer H (2016). Predicting malignant nodules from screening CT scans. J Thorac Oncol.

[14] Dennie C, Thornhill R, Sethi-Virmani V, Souza CA, Bayanati H, Gupta A, Maziak D (2016). Role of quantitative computed tomography texture analysis in the differentiation of primary lung cancer and granulomatous nodules. Quant Imaging Med Surg.

[15] Digumarthy SR, Padole AM, Lo Gullo R, Singh R, Shepard J-AO, Kalra MK (2018). CT texture analysis of histologically proven benign and malignant lung lesions. Medicine (Baltimore).

[16] Chae H-D, Park CM, Park SJ, Lee SM, Kim KG, Goo JM (2014). Computerized texture analysis of persistent part-solid ground-glass nodules: differentiation of preinvasive lesions from invasive pulmonary adenocarcinomas. Radiology..

[17] Cho YJ, Kim WS, Choi YH, Ha JY, Lee S, Park SJ (2019). Computerized texture analysis of pulmonary nodules in pediatric patients with osteosarcoma: differentiation of pulmonary metastases from non-metastatic nodules. PLoS One.

[18] Altenbernd J, Wetter A, Umutlu L, Hahn S, Ringelstein A, Forsting M, Lauenstein T (2016). Dual-energy computed tomography for evaluation of pulmonary nodules with emphasis on metastatic lesions. Acta Radiol.

[19] McCollough CH, Leng S, Yu L, Fletcher JG (2015). Dual- and multi-energy CT: principles, technical approaches, and clinical applications. Radiology.

[20] Deniffel D, Sauter A, Dangelmaier J, Fingerle A, Rummeny EJ, Pfeiffer D (2019). Differentiating intrapulmonary metastases from different primary tumors via quantitative dual-energy CT based iodine concentration and conventional CT attenuation. Eur J Radiol.

[21] Zhang Y, Cheng J, Hua X, Yu M, Xu C, Zhang F (2016). Can spectral CT imaging improve the differentiation between malignant and benign solitary pulmonary nodules?. PLoS One.

[22] Große Hokamp N, Gupta A, Gilkeson RC (2019). Stratification of pulmonary nodules using quantitative iodine maps from dual-energy computed tomography. Am J Respir Crit Care Med.

[23] Traverso A, Wee L, Dekker A, Gillies R (2018). Repeatability and reproducibility of Radiomic features: a systematic review. Int J Radiat Oncol Biol Phys.

[24] Swensen SJ, Brown LR, Colby TV, Weaver AL (1995). Pulmonary nodules: CT evaluation of enhancement with iodinated contrast material. Radiology..

[25] Knöss N, Hoffmann B, Krauss B, Heller M, Biederer J (2011). Dual energy computed tomography of lung nodules: differentiation of iodine and calcium in artificial pulmonary nodules in vitro. Eur J Radiol.

[26] Callister MEJ, Baldwin DR, Akram AR, Barnard S, Cane P, Draffan J (2015). British Thoracic Society guidelines for the investigation and management of pulmonary nodules. Thorax.

[27] Ost DE, Gould MK (2012). Decision making in patients with pulmonary nodules. Am J Respir Crit Care Med.

[28] Schmid-Bindert G, Henzler T, Chu TQ, Meyer M, Nance JW, Schoepf UJ (2012). Functional imaging of lung cancer using dual energy CT: how does iodine related attenuation correlate with standardized uptake value of 18FDG-PET-CT?. Eur Radiol.

[29] Iwano S, Ito R, Umakoshi H, Ito S, Naganawa S (2015). Evaluation of lung cancer by enhanced dual-energy CT: association between three-dimensional iodine concentration and tumour differentiation. Br J Radiol.

[30] Gao C, Xiang P, Ye J, Pang P, Wang S, Xu M (2019). Can texture features improve the differentiation of infiltrative lung adenocarcinoma appearing as ground glass nodules in contrast-enhanced CT?. Eur J Radiol.

[31] Choe J, Lee SM, Do K-H, Lee JB, Lee J-G, Seo JB (2019). Prognostic value of radiomic analysis of iodine overlay maps from dual-energy computed tomography in patients with resectable lung cancer. Eur Radiol.

