# Supplementary material for: Texture analysis of iodine maps and conventional images for k-nearest neighbor classification of benign and metastatic lung nodules
Source: Cancer Imaging. 2021 Jan 26;21:17. doi: 10.1186/s40644-020-00374-3 (PMC7836145; doi:10.1186/s40644-020-00374-3)
Supplement: Supplementary file 1 — Additional file 1 Supplemental Table 1. Calculation of first order texture features. Xi = signal intensity of a voxel; N = number of voxels in the region of interest (ROI). pj = probability of intensity range j in the ROI; Ng = the number of discretized intensity values in the ROI. Supplemental Table 2. Calculation of mutual information (MI), F1-score, diagnostic accuracy, sensitivity and specificity. [file 40644_2020_374_MOESM1_ESM.docx]

**Supplemental digital content**

| Feature | Formula |
| --- | --- |
| *Mean* | $\mu=\frac{1}{N}\sum_{i=1}^{N} X_{i}$ |
| *Standard Deviation* | $\sqrt{\frac{1}{N}\sum_{i=1}^{N} \left( X_{i}-\mu\right)^{2}}$ |
| *Entropy* | $-\sum_{j=1}^{Ng} p_{j}.\log p_{j}$ |
| *Kurtosis* | $\frac{\frac{1}{N}\sum_{i=1}^{N} \left( X_{i}-\mu\right)^{4}}{\left( \frac{1}{N}\sum_{i=1}^{N} \left( X_{i}-\mu\right)^{2} \right)^{2}}-3$ |
| *Mean of the Positive Pixels* | $\frac{1}{N}\sum_{i=1}^{N} X_{i} , \forall X_{i}>0$ |
| *Skewness* | $\frac{\frac{1}{N}\sum_{i=1}^{N} \left( X_{i}-\mu\right)^{3}}{\left( \frac{1}{N}\sum_{i=1}^{N} \left( X_{i}-\mu\right)^{2} \right)^{\frac{3}{2}}}$ |
| *Uniformity* | $\frac{1}{\mathrm{Ng}}\sum_{j=1}^{Ng} {p_{j}}^{2}$ |
| *Uniformity of the positive pixels* | $\frac{1}{\mathrm{Ng}}\sum_{j=1}^{Ng} {p_{j}}^{2}, {\forall X}_{i}>0$ |

**Supplemental table 1:** Calculation of first order texture features. $X_{i}$ = signal intensity of a voxel; N = number of voxels in the region of interest (ROI). $p_{j}$ = probability of intensity range j in the ROI; Ng = the number of discretized intensity values in the ROI.

| Feature | Formula |
| --- | --- |
| Mutual information (MI) | $MI= \sum_{x,y} p_{xy}\log\frac{p_{xy}}{p_{x}p_{y}}$ |
| F1-Score | $\frac{2TP}{(2TP+FN+FP)}$ |
| Accuracy | $\frac{TP+TN}{(TP+FN+FP+TN)}$ |
| Sensitivity | $\frac{TP}{\left( TP+FN \right)}$ |
| Specificity | $\frac{TN}{(TN+FP)}$ |

**Supplemental table 2:** Calculation of mutual information (MI), F1-score, diagnostic accuracy, sensitivity and specificity.
